# Iodine Status of Women of Reproductive Age in Sierra Leone and Its Association with Household Coverage with Adequately Iodized Salt

**DOI:** 10.3390/nu8020074

**Published:** 2016-02-03

**Authors:** Fabian Rohner, James P. Wirth, Bradley A. Woodruff, Faraja Chiwile, Hannah Yankson, Fatmata Sesay, Aminata S. Koroma, Nicolai Petry, Solade Pyne-Bailey, Elisa Dominguez, Roland Kupka, Mary H. Hodges, Mercedes de Onis

**Affiliations:** 1 GroundWork, Crans-près-Céligny 1299, Switzerland; james@groundworkhealth.org (J.P.W.); woody@groundworkhealth.org (B.A.W.); nico@groundworkhealth.org (N.P.); 2 UNICEF, Freetown, Sierra Leone; fchiwile@unicef.org; 3 World Health Organization, Freetown, Sierra Leone; yanksonh@who.int; 4 Helen Keller International, Freetown, Sierra Leone; fsesay@hki.org (F.S.); mhodges@hki.org (M.H.H.); 5 Ministry of Health and Sanitation, Freetown, Sierra Leone; shamitamin@gmail.com (A.S.K.); soeddoal@yahoo.com (S.P.-B.); 6 World Health Organization West Africa, Ouagadougou, Burkina Faso; domingueze@who.int; 7 UNICEF Headquarters, New York, NY 10017, USA; rkupka@unicef.org; 8 World Health Organization Headquarters, 1211 Geneva, Switzerland; deonism@who.int

**Keywords:** iodine status, salt iodization, salt iodine levels, household equity, Sierra Leone

## Abstract

Salt iodization programs are a public health success in tackling iodine deficiency. Yet, a large proportion of the world’s population remains at risk for iodine deficiency. In a nationally representative cross-sectional survey in Sierra Leone, household salt samples and women’s urine samples were quantitatively analyzed for iodine content. Salt was collected from 1123 households, and urine samples from 817 non-pregnant and 154 pregnant women. Household coverage with adequately iodized salt (≥15 mg/kg iodine) was 80.7%. The median urinary iodine concentration (UIC) of pregnant women was 175.8 µg/L and of non-pregnant women 190.8 µg/L. Women living in households with adequately iodized salt had higher median UIC (for pregnant women: 180.6 µg/L *vs.* 100.8 µg/L, respectively, *p* < 0.05; and for non-pregnant women: 211.3 µg/L *vs.* 97.8 µg/L, *p* < 0.001). Differences in UIC by residence, region, household wealth, and women’s education were much smaller in women living in households with adequately iodized salt than in households without. Despite the high household coverage of iodized salt in Sierra Leone, it is important to reach the 20% of households not consuming adequately iodized salt. Salt iodization has the potential for increasing equity in iodine status even with the persistence of other risk factors for deficiency.

## 1. Introduction

Even in its milder forms, iodine deficiency has important effects on the cognitive development and growth of the fetus and the young child [1,2], as well as on cognitive performance in school-aged children [3]. During adulthood, the consequences of iodine deficiency, albeit less profound than for earlier life stages, include hypothyroidism and goiter [4].

Although increased iodization of household salt has reduced iodine deficiency and its disorders globally in the past two decades, an estimated 1.8 billion people in 2011 were at risk of iodine deficiency globally [5]. These estimates are based on the proportion of school-aged children with urinary iodine concentrations below 100 µg/L. In 2015, almost 36 million infants remain “unprotected”; receiving insufficient iodine during their fetal stage and early infancy [6].

In the vast majority of West African countries, including Sierra Leone, data on iodine status are mostly from the early 2000’s. Sierra Leone’s most recent nationally representative data were collected in 2003 [7]. In that survey, the median urinary iodine concentration (UIC) in school-going children was 158 µg/L, which is considered adequate according to the World Health Organization (WHO) [8]. However, important differences across the districts were reported, with those districts in the Northern region having lower median UIC. In addition, the net primary school enrolment ratio in the period 2008–2012 was about 75% [9] and was likely lower in 2003. As a result, the UIC estimate from the 2003 survey may have been an overestimate because children not attending school were excluded, and these children may be at higher risk of iodine deficiency. Furthermore, estimating a population’s iodine status by sampling school-aged children has recently been challenged, since school-age children are not the primary target groups of iodine interventions [4]. The primary beneficiaries are unborn and very young children and, thus, assessment surveys should measure UIC in pregnant and non-pregnant women [10,11,12,13].

In 1994, the Government of Sierra Leone mandated that all salt imported to Sierra Leone be iodized at 35 mg/kg [7]. Since then, assessments have demonstrated a steady increase in the proportion of salt which contains iodine, from 23% in 2000 to 80% in 2013 [14,15]. However, because these assessments used only the rapid salt testing kit, they provide only qualitative information which cannot estimate levels of iodization adequacy [16]. 

The 2013 Sierra Leone Micronutrient Survey (SLMS) quantitatively measured salt iodine content and household coverage with adequately iodized salt. The survey also measured the iodine status of Sierra Leonean pregnant women, non-pregnant non-lactating women, and non-pregnant lactating women.

## 2. Materials and Methods

### 2.1. Study Design and Participants

The SLMS was a cross-sectional survey conducted in November and December 2013. It was designed to yield usable results for each of the two strata (urban and rural). Two- or three-stage sampling was carried out, depending on the target group. Within each stratum, 30 clusters were selected with probability proportionate to size. In each selected cluster, 24 households were selected using simple random sampling. Within a household, all pregnant women were enrolled. An additional stage of sampling selected one non-pregnant woman using a Kish table [10].

### 2.2. Data Collection Procedures

A household questionnaire was administered to the household head or knowledgeable adult in the household. At the end of the household questionnaire, the interviewer asked the respondent to provide a small specimen of household salt for quantitative testing of iodine. Following the household questionnaire, individual questionnaires containing modules on socio-demographic variables, water, sanitation and hygiene, woman and child dietary diversity, and antenatal care were administered to selected women in Krio, Themne, or English, as appropriate. Finally, women were asked to provide a urine sample in a pre-labelled urine container.

Salt iodine content was analyzed quantitatively using the colorimetric method on the iCheck Iodine™ analyzer (Bioanalyt GmbH, Teltow, Germany; [16]). As a quality control measure, every 10th specimen was reanalyzed, and non-concordant values reassessed. Overall coefficient of variation was well below 5% between two technical replicates.

Urinary iodine concentration was determined using the ammonium persulfate/Sandell-Kolthoff reaction method [17] conducted at the newly-established iodine laboratory at the Noguchi Memorial Institute for Medical Research, Ghana. Technicians assessed the concentration of each specimen twice, and the mean of the two results was used as the specimen concentration. Internal quality control materials labeled as low, medium and high were run with specimens. Results from an analytical run were rejected if the value from the internal quality control material was not within the acceptable range.

### 2.3. Case Definitions

Salt iodine concentrations were categorized into five categories: 0 mg/kg, 1–14 mg/kg, 15–34 mg/kg, 35–49 mg/kg, and ≥50 mg/kg. Adequately iodized salt was defined as salt with an iodine concentration ≥15 mg/kg. This dichotomization is not in full agreement with internationally recommended guidelines [8], but because the national legislation “expects” salt iodine levels between 15 and 50 mg/kg at the retail level and 80–100 mg/kg at the factory level [18], defining adequacy as concentrations ≥15 mg/kg is justified to include higher concentrations that may occur from proper adherence to salt iodization standards at the factory level.

Different cut-offs are used to define deficient populations depending on women’s pregnancy and lactation status. Individual women are not classified as deficient or sufficient because an individual’s spot urinary iodine concentration can vary considerably from day to day and thus cannot accurately measure deficiency [19]. The population of non-pregnant, non-lactating women was classified using median UIC as follows [8]: <20 µg/L (severe deficiency), 20–49 µg/L (moderate deficiency), 50–99 µg/L (mild deficiency), 100–199 µg/L (adequate), 200–299 µg/L (above requirements), ≥300 µg/L (excessive). Deficiency in pregnant women was classified according to the median UIC as follows: <150 µg/L (inadequate), 150–249 µg/L (adequate), 250–499 µg/L (more than adequate, ≥500 µg/L (excessive). To produce maps of iodine deficiency, median iodine concentrations for non-pregnant *non-lactating* and *lactating* women were calculated at the cluster level. The proportion of women with UIC less than and equal to or greater to 100 μg/L in each cluster was calculated to illustrate areas of iodine deficiency, sufficiency, and excess.

### 2.4. Data Management and Statistical Analysis

All data were doubly entered using a pre-programmed data-entry screen in CSPro v. 5.0 (U.S. Census Bureau, Washington, DC, USA). Laboratory data were doubly entered into Microsoft Excel, version 2010. Data analysis was done using SPSS version 23 with the complex survey module. For most analyses, standardized statistical weights calculated separately for each target group accounted for the unequal selection probability in the two strata. Histograms and the Kolmogorov-Smirnov test were used to check for the normality of the distribution of numeric data values. The statistical precision of estimates of prevalence rates and means were assessed using 95% confidence limits which were calculated accounting for the complex sampling.

The median UIC was calculated for each target group overall and for subgroups in order to judge population iodine status against WHO criteria [8]. However, in order to judge the statistical precision of apparent differences among subgroups, a square root transformation of the UIC values created a variable which was normally distributed. ANOVA was then used to calculate p values for apparent differences in geometric means among subgroups. These p values appropriately accounted for the statistical weighting and complex sampling. For comparisons of categorical data, the adjusted chi^2^ test was applied.

Using data on household characteristics and assets, principal component analysis was used to calculate an index of household wealth, used to subsequently classify households into wealth quintiles [20,21].

Geographic analysis techniques were employed to geographically present the distribution of adequately iodized salt and median UIC. Specifically, cluster-specific estimates of the prevalence of adequately iodized salt and the median UIC were linked to latitude and longitude coordinates for each cluster, and inverse distance weighting (IDW) was used to estimate these distributions [22]. For the IDW procedure, a distance coefficient P, which specifies the rate of influence as distance from the point increases, was set to 5.0 due to the relatively large distance between many of the clusters selected for the SLMS. Geographic analysis was conducted using the interpolation function of Quantum GIS 2.6.

### 2.5. Ethics and Consent

The survey protocol was approved by the Office of the Sierra Leone Ethics and Scientific Review Committee, Directorate of Training, Non-Communicable Diseases and Research, Connaught Hospital, Ministry of Health and Sanitation. Oral consent was sought for the household interview, and written informed consent was requested for urine sampling. To compensate for the sampled salt, participating households were provided with 250 g of adequately iodized salt.

## 3. Results

### 3.1. Response Rates and Respondents’ Characteristics

The household response rate was 97.0%, leading to completed household interviews of 1354 households. Of these 1160 (85.7%) provided a salt sample, but for only 1123 (96.8%) samples was the quantity sufficient for laboratory analysis. From participating households, 945 non-pregnant women 15–49 years of age were randomly selected for inclusion in the survey. Of these women, 817 (86.5%) completed the interview and had a valid result for UIC. In addition, 178 eligible pregnant women in selected households were asked to participate in the study, and 154 (86.5%) completed the questionnaire and had a valid UIC result.

The median household size was 5.5 members, 60.4% of households were located in rural areas, 72.7% were male-headed, 99.8% used “natural” cooking fuel (e.g., charcoal or wood), 62.2% had “unimproved” sanitation facilities, and 76.5% drank safe water.

Overall, 787 (83%) of non-pregnant women had been pregnant previously. Among these, 33.2% were lactating at the time of the survey. The mean age of non-pregnant women was 27.7 years and of pregnant women was 23.4 years. No formal school attendance was reported by 55.5% of non-pregnant and 52.8% of pregnant women. In addition, 68.6% of non-pregnant and 79.8% of pregnant women were illiterate as measured by a simple reading test administered during the interview.

Only 27.3% of all responding women, pregnant and non-pregnant, reported ever having heard about iodized salt, and among these women, 31.4% were able to provide a correct answer of reported benefits of iodized salt (prevention of goiter or iodine deficiency, improvement of health or intelligence).

### 3.2. Household Coverage with Iodized Salt

The vast majority (87.0%) of salt collected was not in the original package. Despite this fact, the proportion of salt samples which were adequately iodized as per international standards was quite high; 77.5% of samples in the range of 15–40 mg/kg, see Table 1. Using a simplified definition for adequate iodization (*i.e.*, iodine content ≥15 mg/kg), 80.7% (95% CI: 73.1, 86.5) of salt samples were adequately iodized. Urban and wealthier households had a higher coverage of adequately iodized salt than rural and poorer households. There were also marked differences among regions, with the Northern region having a considerably lower coverage of adequately iodized salt than the other three regions.

**Figure 1:**
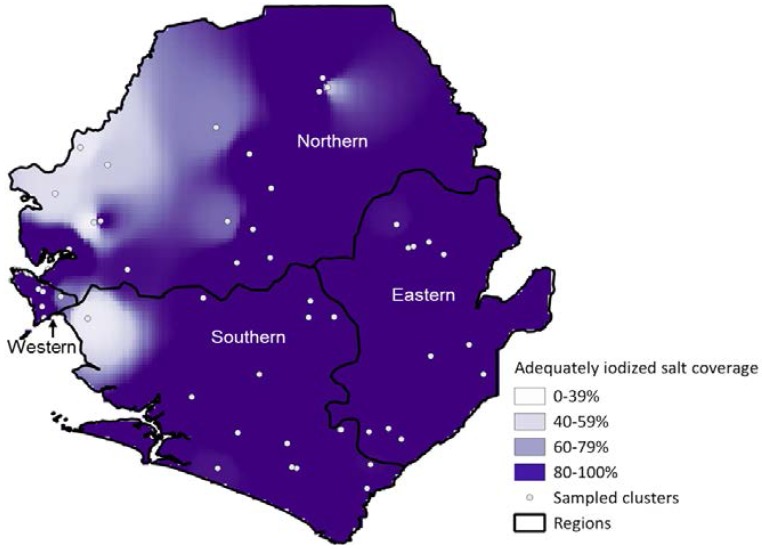
Geographic distribution of coverage of adequately iodized salt (≥15 mg/kg), Sierra Leone, 2013.

These geographic differences become more evident when looking at the interpolated map of the coverage of adequately iodized salt (Figure 1). In several areas of the Northern region and a smaller portion of the Southern region, coverage is considerably lower than elsewhere in the country.

**Table 1:** Weighted distribution of household salt iodine levels and of adequately iodized salt, Sierra Leone, 2013.

Characteristic	*n*	% ^a^	(95% CI) ^b^	ANOVA *p* Value ^c^
Results of quantitative analysis, by iodization adequacy				
Not iodized (0 mg/kg)	4	0.3	(0.1, 0.9)	-
Insufficiently iodized (<15 mg/kg)	201	19.0	(13.2, 26.6)	
Adequately iodized (≥15 mg/kg) ^d^	923	80.7	(73.1, 86.5)	
15–40 mg/kg	886	77.4	(70.1, 83.4)	
41–50 mg/kg	30	2.6	(1.7, 4.0)	
>50 mg/kg	7	0.6	(0.2, 1.8)	
Adequately iodized ^d^, by residence				
Urban	475	88.0	(81.3, 92.5)	< 0.05
Rural	448	76.2	(64.5, 84.9)	
Adequately iodized ^d^, by region				
Eastern	204	89.3	(84.5, 92.7)	< 0.05
Northern	262	68.7	(53.3, 80.8)	
Southern	236	84.4	(65.3, 94.0)	
Western	221	88.3	(78.7, 93.9)	
Adequately iodized ^d^, by wealth quintile				
Lowest	159	73.6	(56.6, 85.7)	< 0.01
Second	163	75.0	(61.4, 84.9)	
Middle	172	80.0	(71.2, 86.6)	
Fourth	180	83.1	(74.6, 89.1)	
Highest	223	94.0	(89.9, 96.4)	

^a^ Percentages weighted for unequal probability of selection; ^b^ CI = confidence interval, calculated taking into account the complex sampling design; ^c^ Chi-square *p*-value < 0.05 indicates that the proportion in at least one subgroup is statistically significantly different from the values in the other subgroups; ^d^ For the presentation of these sub-group results, “adequately iodized” has been defined as containing ≥15 mg/kg iodine.

### 3.3. Pregnant Women

The median UIC among pregnant women in Sierra Leone is 175.8 µg/L, which is considered adequate. Because the SLMS was a household-based survey, the sample of pregnant women and consequently, the number of UIC results from this group is small. As a result, analysis of sub-groups may have limited use because of the severely limited precision of sub-group specific estimates. Nonetheless, some key findings are presented in Table 2. For several sub-groups, such as women age 25–34 years of age, rural women, women in the Northern region, women who have never attended school, women in the lowest and highest wealth quintiles, and women living in households without adequately iodized salt, the median UIC are below the threshold for adequate iodine status. In no sub-group does the median UIC exceed recommended levels (*i.e.*, ≥250 µg/L).

Pregnant women living in households with adequately iodized salt at the time of the survey had statistically significantly higher UIC than women in households with inadequately iodized salt. Although not statistically significant, the median UIC progressively increases with educational level. In contrast, there is no such progressive change in median UIC with wealth index or woman’s age.

**Table 2:** Median urinary iodine in pregnant women, Sierra Leone 2013.

Characteristic	*n* ^a^	Median Urinary Iodine (µg/L)	ANOVA *p* Value ^b^
Adequately iodized salt in household ^c^			
Yes	109	180.6	< 0.01
No	21	100.8	
Residence			
Urban	57	179.4	0.33
Rural	97	148.3	
Region			
Eastern	32	201.7	0.29
Northern	52	138.1	
Southern	39	150.5	
Western	31	207.2	
Women’s education			
Never attended school	82	142.0	0.12
Completed primary school or less	31	168.3	
Some/completed secondary or more	41	195.1	
Wealth quintile			
Lowest	28	136.5	0.57
Second	38	175.8	
Middle	29	189.0	
Fourth	31	178.6	
Highest	22	134.6	
Age group (in years)			
15–24	93	183.2	< 0.05
25–34	50	137.3	
35+	8	209.0	

^a^ Numbers are un-weighted numbers in each subgroup; the sum of subgroups may not equal the total because of missing data. Total sample size = 154; ^b^ ANOVA p value for differences in weighted geometric mean; a *p* value < 0.05 indicates that the geometric mean in at least one subgroup is statistically significantly different from the values in the other subgroups; ^c^ Adequately iodized salt >15 mg/kg.

### 3.4. Non-Pregnant Women

At the national level, the median UIC indicate adequate iodine status for both non-lactating non-pregnant women (median UIC: 203.3 µg/L) and lactating non-pregnant women (median UIC: 175.6 µg/L).

In contrast to pregnant women, the median UIC in nearly all subgroups of non-pregnant non-lactating women (Table 3) and non-pregnant lactating (Table 4) were substantially above the threshold of 100 µg/L which defines iodine sufficiency in these population groups (Table 3). Only lactating women residing in households where salt was inadequately iodized had a geometric mean UIC below 100 µg/L. For several sub-groups of non-pregnant non-lactating and lactating women, median UIC were in the range above requirements, albeit mostly just above the threshold of 200 µg/L.

Similar to pregnant women, non-pregnant women living in households with adequately iodized salt had substantially higher UIC than women in households without adequately iodized salt. In contrast to pregnant women, and probably due to the larger sample size, UIC was also statistically significantly associated with age, urban residence, region of residence, educational level, and household wealth in non-lactating and/or lactating women. In non-lactating women, there was a progressive increase in the geometric mean UIC with educational level and household wealth, but not with age.

**Table 3:** Weighted geometric mean urinary iodine in non-pregnant non-lactating women 15–49 years, Sierra Leone 2013.

Characteristic	*n* ^a^	Median Urinary Iodine (µg/L)	ANOVA *p* Value ^b^
Adequately iodized salt in household ^c^			
Yes	401	217.2	<0.001
No	88	122.8	
Residence			
Urban	328	224.2	<0.001
Rural	243	174.8	
Region			
Eastern	105	190.5	0.26
Northern	167	192.9	
Southern	143	184.7	
Western	156	222.8	
Women’s education			
Never attended school	303	174.8	<0.001
Completed primary school or less	56	205.8	
Some/completed secondary or more	211	235.2	
Wealth quintile			
Lowest	91	173.0	<0.001
Second	84	167.7	
Middle	116	195.6	
Fourth	120	205.8	
Highest	142	253.3	
Age (in years)			
15–19	110	252.8	<0.05
20–24	103	189.5	
25–29	88	164.8	
30–34	78	204.3	
35–39	73	207.9	
40–44	63	192.2	
45–49	40	174.8	

^a^ Numbers are un-weighted numbers in each subgroup; the sum of subgroups may not equal the total because of missing data. Total sample size = 571; ^b^ ANOVA p value for differences in weighted geometric mean; a *p* value < 0.05 indicates that the geometric mean in at least one subgroup is statistically significantly different from the values in the other subgroups; ^c^ Adequately iodized salt >15 mg/kg.

**Table 4:** Weighted geometric mean urinary iodine in non-pregnant lactating women 15–49 years, Sierra Leone 2013.

Characteristic	*n* ^a^	Median Urinary Iodine (µg/L)	ANOVA *p* Value ^b^
Adequately iodized salt in household ^c^			
Yes	143	196.8	<0.001
No	39	75.6	
Residence			
Urban	81	210.1	0.38
Rural	139	165.0	
Region			
Eastern	59	187.9	<0.05
Northern	80	140.6	
Southern	55	185.5	
Western	26	220.8	
Women’s education			
Never attended school	140	168.7	0.68
Completed primary school or less	33	164.8	
Some/completed secondary or more	47	205.8	
Wealth quintile			
Lowest	63	140.4	0.62
Second	51	172.0	
Middle	44	203.3	
Fourth	34	175.6	
Highest	27	194.2	
Age (in years)			
15–19	46	190.8	<0.05
20–24	45	211.5	
25–29	51	140.6	
30–34	30	140.4	
35–39	22	125.1	
40–44	9	330.1	
45–49	5	171.6	

^a^ Numbers are un-weighted numbers in each subgroup; the sum of subgroups may not equal the total because of missing data. Total sample size = 220; ^b^ ANOVA *p* value for differences in weighted geometric mean; a *p* value < 0.05 indicates that the geometric mean in at least one subgroup is statistically significantly different from the values in the other subgroups; ^c^ Adequately iodized salt >15 mg/kg.

Stratified analysis investigated confounding by household salt iodization as a potential cause of the apparent associations in non-pregnant women between UIC and age, residence, region, education and household wealth (Table 5). Differences in geometric mean UIC between women in urban and rural households and households with different wealth indices are not entirely explained by the adequacy of household salt iodization, as demonstrated by persisting statistically significant associations between UIC and these factors among women with adequate salt iodization. In contrast, differences in UIC by region and educational status are not statistically significant when household salt is adequately iodized. Overall, for all five factors, the differences among subgroups of women with inadequately iodized salt are substantially greater than the corresponding differences in women with adequately iodized salt.

**Table 5:** Weighted median urinary iodine in non-pregnant women (15–49 years), by various demographic characteristics and adequacy ^a^ of salt iodization, Sierra Leone 2013.

	Non-Pregnant Women in Households with Inadequate Salt Iodine			Non-Pregnant Women in Households with Adequate Salt Iodine		
Characteristic	*n* ^b^	Median Urinary Iodine (µg/L)	ANOVA Test *p* Value ^c^	*n* ^b^	Median Urinary Iodine (µg/L)	ANOVA Test *p* Value ^c^
Residence						
Urban	44	143.3	0.05	301	228.7	<0.01
Rural	87	83.4		264	189.5	
Region						
East	16	157.9	<0.05	107	199.0	0.36
North	69	83.3		157	208.2	
South	29	74.9		151	199.7	
West	17	205.9		150	222.7	
Women’s education						
Never attended school	86	74.9	<0.001	295	197.6	0.16
Completed primary school or less	23	122.8		62	201.9	
Some/completed secondary or more	22	221.3		207	222.8	
Wealth index						
Lowest	38	58.6	<0.001	98	187.5	<0.05
Second	29	86.1		87	180.5	
Middle	27	211.8		108	195.2	
Fourth	27	137.8		104	216.5	
Highest	8	289.0		151	247.7	
Age (in years)						
15–19	24	123.6	0.65	110	252.8	0.13
20–24	21	116.3		110	212.3	
25–29	31	92.6		99	183.9	
30–34	17	142.0		70	199.7	
35–39	14	83.3		77	203.3	
40–44	11	88.4		51	232.9	
45–49	6	72.8		31	182.8	

^a^ Adequately iodized salt > 15mg/kg; ^b^ Numbers are unweighted numbers in each subgroup; the sum of subgroups may not equal the total because of missing data. Total sample size = 817; ^c^ ANOVA *p* value for differences in weighted geometric mean.

**Figure 2:**
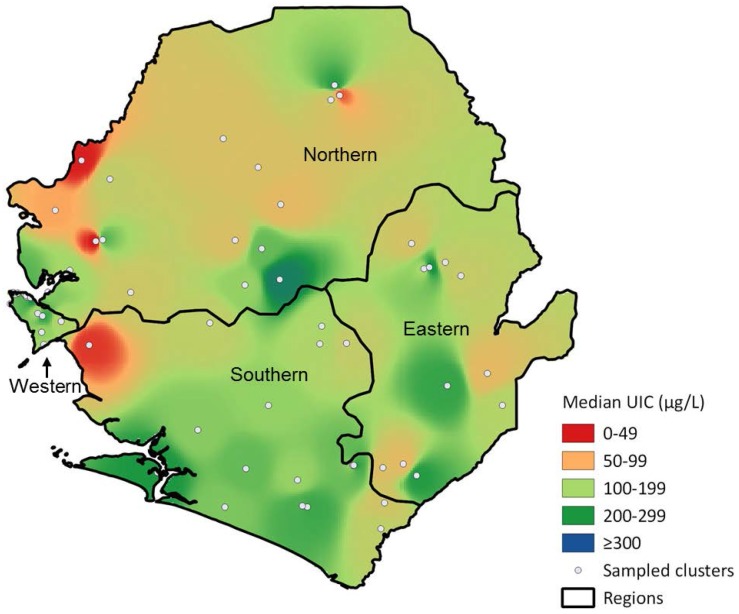
Geographic distribution of median urinary iodine concentrations among non-pregnant women aged 15–49 years, Sierra Leone, 2013.

Figure 2 shows the geographic distribution of median UIC, and the comparison with Figure 1 shows considerable overlap between the levels of coverage with adequately iodized salt and median UIC. With the exception of the West region, in which the capital of Sierra Leone is located, poorer coverage with iodized salt and lower median UIC are concentrated in western parts of Sierra Leone. A few clusters had median UIC ≥300 µg/L but they do not appear in the map because of the IDW technique used and their vicinity to cluster in the “adequate” or “above adequate” range.

## 4. Discussion

### 4.1. Household Coverage with Adequately Iodized Salt

Iodine status has clearly improved in Sierra Leone since the year 2000 when only 23% of households had iodized salt as measured using qualitative test kits [7,23]. Despite this progress, the country has not yet achieved universal salt iodization (USI) according to the definitions of the WHO and other organizations; one criteria for USI is that more than 90% of households are to be consuming adequately iodized salt [8]. As seen in the geographic distribution of coverage, relatively small areas with substantially lower coverage bring the overall coverage down. Our results are in good agreement with those from the Demographic and Health Survey (DHS) conducted in 2013, which had a much larger sample size but analyzed salt qualitatively using test kits [14]. Those districts where SLMS clusters had low coverage with adequately iodized salt are similar to those which had the lowest coverage with iodized salt in the 2013 DHS: Bonthe, Kambia, Moyamba, Port Loko and Western rural districts. These areas are coastal zones, where salt harvesting activities are carried out by the population without subsequent salt iodization.

Salt continues to be harvested on a small scale in Sierra Leone, and the majority of other table salt consumed is imported. While the main salt producers in West Africa are Senegal and Ghana [24], it appears from trade figures that 75% of the salt imported to Sierra Leone originates from India [25]. But this is from trade figures and no comprehensive market assessment has been conducted so far, so variations may occur. That said, the quality of imported iodized salt appears to have improved over the past decade. The Sierra Leone Standards Bureau enacted legislation in 2011 defining standards for imported iodized salt and establishing a monitoring system.

### 4.2. Iodine Status among Non-Pregnant and Pregnant Women

The West of Sierra Leone’s Northern and Southern regions have the lowest coverage of adequately iodized salt. Areas with the lowest median UIC show geographic overlap. In addition, women from households with adequately iodized salt have higher geometric mean UIC, demonstrating a positive association between consumption of iodized salt and iodine sufficiency.

Other variables, such as region of residence, educational level, and household wealth, were also associated with UIC; however, these associations are largely, albeit not completely, explained by differences in the coverage of adequately iodized salt. Differences between subgroups were much smaller in households with adequately iodized salt implying that provision of iodized salt acts as an equalizer. For example, in households in which salt was inadequately iodized, the geometric mean UIC for women in rural households was below the 100 µg/L cut-off defining iodine deficient populations, and the geometric mean UIC in urban women demonstrated iodine sufficiency. When adequately iodized salt was used in the household, both urban and rural women demonstrated iodine sufficiency, and difference in geometric mean between urban and rural women was substantially lower. As a result, although dietary or other risk factors produce lower UIC in rural women, women in the North and South regions, women who never attended school, and women in poorer households, these risk factors are largely mitigated by provision of iodized salt, resulting in much greater equity in iodine status throughout the population of non-pregnant Sierra Leonean women. This highlights the importance of extending the coverage of adequately iodized salt to all segments of the population, especially to the most disadvantaged households in which UIC is lowest.

The number of pregnant women included in the survey is small and thus, subgroup analyses must be interpreted with caution. Nonetheless, the national level median UIC of 176 µg/L in this target group is within the range that defines adequate iodine status. In the past, iodine assessment surveys have often targeted children in schools as a proxy population for women of reproductive age and pregnant women [4,12]; however, this results in highly biased samples in populations where school attendance is far from universal. Pregnant women are probably the most appropriate population for assessment because iodine supply is most critical to their unborn children. Nonetheless, because they comprise a relatively small proportion of the population, household surveys rarely recruit a large number of pregnant women. For assessments of population iodine status which may include stratified or subgroup analyses, non-pregnant women of reproductive age may serve as a more appropriate target group. However, a recent analysis of multiple datasets found that even when non-pregnant women showed iodine sufficiency, this was not always true for pregnant women in the same population [12]. As a result, pregnant women should systematically be included in population assessments even if the survey lacks sufficient sample size of pregnant women to calculate stratum- or subgroup-specific estimates with acceptable precision.

### 4.3. Strengths and Limitations of the Data

Quantitative analysis of salt iodine content represents an important strength of this study. Only quantitative analysis allows conclusions about the adequacy of salt iodization. For this reason, it is an important complement to the MICS and DHS surveys that, although having a much larger sample size, can only distinguish between salt containing iodine and salt not containing iodine because the rapid test kits used only detect the presence or absence of iodine in salt samples.

Although random selection of households likely produces less biased estimates than sampling school attendees at schools, as was done in the past, the data presented here cannot be directly compared to previous surveys. Also, household sampling has important consequences on the sample size of pregnant women as described above. In this survey, the number of pregnant women is well below the minimum sample size of 300 recommended for assessment of UIC [8]. In addition, subgroup analyses and cluster-based mapping among non-pregnant women require larger sample sizes. That said, the estimates of iodine status for pregnant and non-pregnant women in this survey are consistent with each other. Moreover, even with this limited sample size, some statistically significant associations between UIC and other factors were detected.

## 5. Conclusions

Although universal salt iodization in Sierra Leone is not yet achieved as measured by household coverage with iodized salt, the national median UIC of both pregnant and non-pregnant women indicate adequate iodine status. Furthermore, salt iodine content and UIC are strongly associated, and adequately iodized salt produces greater equity in iodine status, indicating that salt iodization continues to be a crucial strategy in supplying dietary iodine to the Sierra Leonean population. A few pockets of low coverage with adequately iodized salt still exist, mostly along coastal districts, and these pockets exhibit with lower median UIC. Strengthening efforts to reach these pockets with adequately iodized salt will be important to attain universal salt iodization in the future.

## Abbreviations

The following abbreviations are used in this manuscript:

DHS: Demographic and Health Survey
EVD: Ebola Virus Disease
IDD: Iodine deficiency disorders
MICS: Multiple-Indicator Cluster Survey
SLMS: Sierra Leone Micronutrient Survey
UIC: Urinary iodine concentration
WHO: World Health Organization
